# Evaluation of the Anxiolytic and Antidepressant Activities of the Aqueous Extract from *Camellia euphlebia* Merr. ex Sealy in Mice

**DOI:** 10.1155/2015/618409

**Published:** 2015-11-01

**Authors:** Dongye He, Xitao Wang, Ping Zhang, Xinxing Luo, Xiaoyu Li, Lili Wang, Shuying Li, Yongping Xu

**Affiliations:** ^1^School of Life Science and Biotechnology, Dalian University of Technology, Dalian 116024, China; ^2^Ministry of Education Center for Food Safety of Animal Origin, Dalian University of Technology, Dalian 116620, China; ^3^Dalian SEM Bio-Engineering Technology Co. Ltd., Dalian 116620, China

## Abstract

*Camellia euphlebia* Merr. ex Sealy is a traditional Chinese medicine that has been widely used for improvement of human emotions in the Guangxi Province of southern China. However, there are no studies about the anxiolytic and antidepressant activities of *Camellia euphlebia*. This study evaluated the anxiolytic and antidepressant activities of the aqueous extract from *Camellia euphlebia* (CEE) in mice. We found that administration of 400 mg/kg CEE or 20 mg/kg fluoxetine for 7 days significantly reduced the immobility time in both TST and FST. Oral administration of 100 mg/kg extract or 4 mg/kg diazepam for 7 days significantly increased the percentage of time spent and the number of entries into the open arms of the EPMT. In addition, the time spent by mice in the illuminated side of the LDBT was increased. Furthermore, pretreatment with 400 mg/kg CEE for 7 days significantly elevated the level of 5-HT and DA in the whole brain of mice. These results provide support for the potential anxiolytic and antidepressant activity of *Camellia euphlebia* and contribute towards validation of the traditional use of *Camellia euphlebia* in the treatment of emotional disorders.

## 1. Introduction

Approximately 450 million people worldwide suffer from mental illness or behavioral disorders, which account for 12.3% of the global burden of disease [1]. It is estimated that this percentage will reach 15% by 2020 [2]. Among the many mental illnesses and behavioral disorders, depression and anxiety are the two most prevalent psychiatric disorders [3]. Several classical anxiolytic and antidepressant drugs such as benzodiazepines, monoamine oxidase inhibitors, tricyclic antidepressants, selective serotonin reuptake inhibitors, serotonin-norepinephrine reuptake inhibitors, and noradrenergic and specific serotonergic antidepressants are widely used in clinical practice to treat these disorders. However, treatment by the above-mentioned drugs can also bring undesirable side effects including cardiovascular toxicity, sexual dysfunction, weight gain, and drug interactions [4–6]. Therefore, there is an urgent need for the development of effective anxiolytic and antidepressant therapies without any or at least fewer adverse effects.

In recent years, many traditional Chinese medicinal plants such as* Acanthopanax senticosus* [7],* Acorus calamus* [8],* Albizia julibrissin* [9],* Ginkgo biloba* [10],* Paeonia lactiflora* [11], and* Hypericum perforatum* [12] have been successfully used to prevent or treat anxiety and depression. Thus, traditional Chinese medicines may be effective alternatives for the treatment of psychiatric disorders.

*Camellia euphlebia* Merr. ex Sealy (Theaceae), called Fortune's Yellow Camellia, is an evergreen shrub standing about 2 to 5 m in height, found in the Guangxi Zhuang Autonomous Region of southern China. The leaves of* Camellia euphlebia* are commonly used for the treatment of dysentery, hypertension, diarrhea, faucitis, and irregular menstruation. The extracts of* Camellia euphlebia* were reported to possess anticarcinogenic, antioxidant, hypoglycemic, and hypolipidemic properties [13–16]. Phytochemical studies on the leaf extract have shown the presence of theanine (*γ*-glutamylethylamide), *γ*-aminobutyric acid (GABA), and caffeine, which can exert different degrees of neuroprotection [13, 17–19]. However, the use of the leaf extract of* Camellia euphlebia* (CEE) has yet to be supported by pharmacological data to prove its anxiolytic and depressant effects.

The present study investigated the anxiolytic activities of CEE in mice, at doses of 100, 200, and 400 mg/kg/day, using the light-dark box test (LDBT) and the elevated plus-maze test (EPMT), and investigated the antidepressant activities of CEE by the forced swimming test (FST) and tail suspension test (TST).

## 2. Materials and Methods

### 2.1. Plant Material and Preparation of the Aqueous Extract

Fresh leaves of* Camellia euphlebia* Merr. ex Sealy were obtained in Guangxi Zhuang Autonomous Region during its flowering period and authenticated by Ph.D. Zhonghhui Ma (Department of Botany Sciences, College of Agriculture, Guangxi University, China). A voucher specimen with number 8109255 has been deposited in the herbarium of the Guangxi Institute of Botany, Chinese Academy of Sciences, China.

The fresh leaves were washed three times with tap water and then dried at 55°C for 6 h in a forced air oven. The dried leaves were ground into a coarse powder with a pulverizer (HC-150T2, Yongkang Lv Ke Food Machinery Company, Zhejiang, China) at room temperature and then further ground through a 200 mesh sieve to obtain a fine powder. Twenty grams of the fine power was extracted twice with 400 mL distilled water (1 : 20) for 2 h at 100°C using an electrical heating jacket (ZNHW, Gongyi Zi Hua Instrument Company, Henan, China) with occasional stirring. Thereafter, the mixture was centrifuged at 10,000 rpm for 10 min and the supernatant was filtered using a 0.22 *μ*m Millipore Filter (Merck Millipore, Billerica, MA, USA) to remove particulate matter. A lyophilized powder with a yield of 19.3% (w/w) was prepared using a freeze dryer (FD-1A-50, Beijing Bo Kang Experimental Medical Instrument Company, Beijing, China) and stored in a sealed bag at −20°C until use.

### 2.2. Animals

Male Kunming mice, weighing 20 to 25 g, were purchased from the Experimental Animal Centre of Dalian Medical University (Dalian, China). The animals were housed with a 12 h light/dark cycle under controlled humidity (50 ± 10%) and temperature (22 ± 2°C) and were allowed free access to food and water. The animals were acclimatized for at least 3 days before they were tested and were randomly assigned to the various experimental groups (*n* = 6 per group). All animal experiments were performed in compliance with the recommendations of the Local Institutional Animal Care and National Act on the use of experimental animals (Beijing, China).

### 2.3. Drugs and Administration

For each test, mice were divided into five groups including vehicle control, positive control, and three experimental groups. Animals in the vehicle control group were administered normal saline (0.9% NaCl solution). In the antianxiety tests, the positive control group was treated with 4 mg/kg diazepam (DZP, Sigma, St Louis, MO) as an anxiolytic drug, while in the antidepression tests, the positive control group was treated with 20 mg/kg fluoxetine (FLU, Patheon, Jallieu Cedex, France) as an antidepressant drug. Mice in the three experimental groups were administered CEE at doses of 100, 200, or 400 mg/kg. All administrations were performed in a volume of 0.4 mL per 25 g body weight. All drugs were administrated only once per day via gastric intubation between 9:30 and 10:30 a.m. for 7 consecutive days. The tests were conducted 1 h after the last drug treatment.

### 2.4. Acute Toxicity Study

Mice (10 per treatment) were treated orally with normal saline (0.4 mL per 25 g) or CEE (500, 1000, 2000, or 4000 mg/kg) between 9:00 and 10:00 a.m. Each intragastric administration was performed after a 6 h fasting period. Mice were observed for toxic symptoms and mortality was calculated after 24 h of the last-treatment [20].

### 2.5. Antianxiety Tests in Mice

#### 2.5.1. Light-Dark Box Test (LDBT)

The light-dark box test was carried out according to the method of Costall et al. [21]. The test apparatus consisted of a plexiglass box (length 45 cm × width 27 cm × height 27 cm) with two compartments with 40% dark area and 60% brightly illuminated white area. Mice were placed individually into the center of the illuminated compartment, facing one of the light areas. The time spent in the light box was recorded for 5 min. Entrance into the light box was regarded as an index of less anxiety [22].

#### 2.5.2. Elevated Plus-Maze Test (EPMT)

The elevated plus-maze test was carried out according to the method described by Carrasco et al. [23]. The test apparatus consisted of two enclosed arms (length 30 cm × width 5 cm × height 15 cm), two open arms (length 30 cm × width 5 cm), and a central platform (5 cm × 5 cm). The maze was elevated 45 cm above the floor level. Mice were placed individually into the center of the maze, facing one of the open arms. The number of open arms entries and the time spent in the enclosed and open arms were recorded for 5 min with a video camera. Increased activity in the open arms was indicative of less anxiety [24]. Entry into an arm was defined when mice placed all four feet into the arm. The maze was cleaned with 10% ethanol solution after each test.

### 2.6. Antidepression Tests in Mice

#### 2.6.1. Forced Swimming Test (FST)

The forced swimming test was based on the method of Porsolt et al. [25]. Briefly, mice were forced to swim individually in a glass cylinder (20 cm × 14 cm) containing fresh water up to a height of 10 cm at 25 ± 1°C. All animals were forced to swim for a 6 min period and the total duration of immobility was recorded during the last 4 min with a video camera. Mice were considered immobile when they floated in the water without struggling and making only those movements necessary to keep their heads above the water.

#### 2.6.2. Tail Suspension Test (TST)

The tail suspension test was conducted as previously described by Steru et al. [26]. Briefly, mice were suspended individually 5 cm above the floor by an adhesive tape placed approximately 1 cm from the tip of the tail. Testing was carried out in a darkened room with minimal background noise. Mice were considered to be immobile only when they hung passively or remained completely motionless. The total duration of immobility was recorded during the final 4 min of the 6 min test and scored by an observer blind to treatment.

### 2.7. Open-Field Test (OFT)

To exclude nonspecific effects of CEE, the spontaneous locomotor activity of mice was measured in the open-field test. The test was carried out on mice according to a slightly modified method [27]. The apparatus consisted of an opaque-plexiglass box (40 cm × 40 cm × 40 cm) with the floor divided equally into 25 squares. Mice were placed individually into the center of the arena and allowed to explore freely. The number of crossings (number of squares crossed by the mouse with the four paws) and the number of rearing (standing on the hind legs) were recorded for 3 min.

### 2.8. Determination of Monoamine Neurotransmitter Levels

After the tail suspension test, mice were sacrificed immediately by decapitation, and the whole brain was quickly removed, frozen in liquid nitrogen, and stored at −70°C for biochemical analysis. The whole brain tissue was homogenized using a Teflon-glass homogenizer. A 30 mg portion of the homogenate was diluted in 300 *μ*L normal saline (0.9%) and then centrifuged at 10000 g for 10 min at 4°C. The levels of 5-HT and DA in the supernatant were measured by ELISA (Nanjing Jiancheng Bioengineering Institute, Nanjing, China). Dispensed antigen standards and samples were added to each well of 96-well plates precoated with primary antibodies. After adding biotin conjugate reagent and enzyme conjugate reagent into each well, the plates were incubated at 37°C for 60 min. Then the plates were rinsed five times with wash solution. Within 10 min of the chromogenic reaction, the absorbance was measured at 450 nm using a microplate reader (TECAN, Sunrise, Männedorf, Austria).

### 2.9. Statistical Analysis

The data were statistically analyzed with one-way analysis of variance (ANOVA) followed by Tukey's *t*-test using GraphPad Prism 5.03 Software (GraphPad Software Inc., San Diego, CA, USA). The results were expressed as the mean ± standard error (S.E.M.). Differences among groups were considered to be statistically significant at *P* < 0.05.

## 3. Results

### 3.1. Acute Toxicity Study

The aqueous extract (500 to 4000 mg/kg) had a high safety profile, as no deaths were observed at the dose levels tested. The behavioral changes observed at high doses of CEE (2000 or 4000 mg/kg) were sedation and hyperventilation (Table 1).

### 3.2. Effects of CEE on the Time Spent in the Illuminated Side of the LDBT

**Figure 1:**
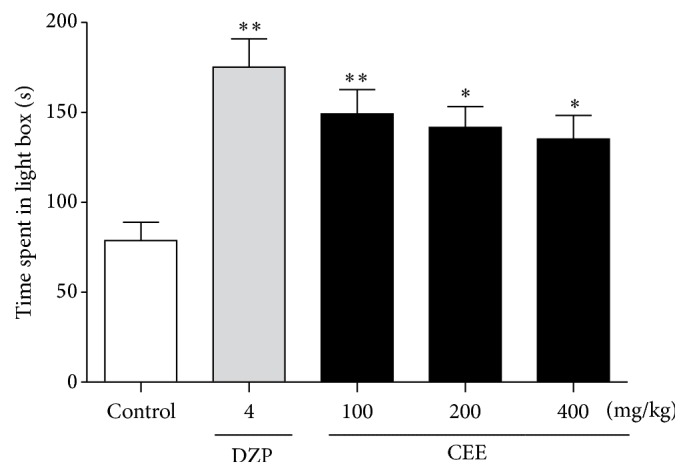
Effects of oral administration of CEE (100, 200, or 400 mg/kg) or diazepam (4 mg/kg) on the time spent in the illuminated side of the light-dark box test in mice. The total time spent in the light box was recorded 1 h after the last administration. Values are presented as mean ± S.E.M. (*n* = 6 per group). ^*∗*^ *P* < 0.05, ^*∗∗*^ *P* < 0.01 as compared with vehicle control.

As is shown in Figure 1, compared with control group, the oral administration of CEE at doses of 100, 200, or 400 mg/kg as well as 4 mg/kg diazepam significantly increased the time spent in the light box by mice [*P* < 0.01, *P* < 0.05, *P* < 0.05, and *P* < 0.01, resp.]. Moreover, in this test, the anxiolytic effects of CEE were dose-dependent with a trend to decline after 7 consecutive days of treatment.

### 3.3. Effects of CEE on Mice Behavior in the EPMT

**Figure 2:**
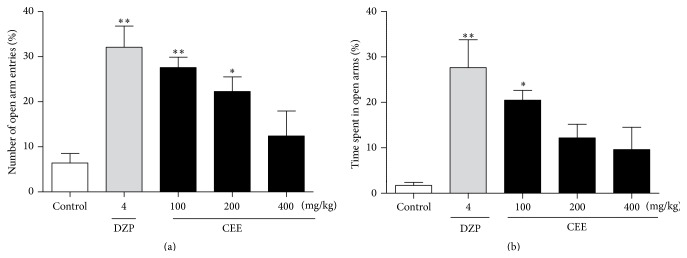
Effects of oral administration of CEE (100, 200, or 400 mg/kg) or diazepam (4 mg/kg) on the behaviors in the elevated plus-maze test in mice. The total time spent in the light box was recorded 1 h after the last administration. (a) The number of open arm entries during the total 5 min test in mice. (b) The time spent in open arms during the total 5 min test in mice. Values are presented as mean ± S.E.M. (*n* = 6 per group). ^*∗*^ *P* < 0.05, ^*∗∗*^ *P* < 0.01 as compared with vehicle control.

The results presented in Figure 2(a) indicate that treating mice with CEE at doses of 100 (*P* < 0.01) or 200 mg/kg (*P* < 0.05) for 7 days significantly increased the percentage of open arm entries by 76.77 and 71.24%, respectively, compared with the control group. However, the difference between the control and the dose of 400 mg/kg was not statistically significant.

Figure 2(b) showed that treatment with CEE at a dose of 100 mg/kg resulted in statistically significant changes in the percentage of time spent in the open arm compared with the control group (*P* < 0.05), while the doses of 200 and 400 mg/kg were not statistically significant. In addition, diazepam (4 mg/kg) as a widely used anxiolytic drug in clinical practice significantly increased the percentage of open arm entries and the percentage of time spent in the open arms in the EPMT, by 80.03 and 93.75%, respectively (*P* < 0.01).

### 3.4. Effects of CEE on the Duration of Immobility in the FST

**Figure 3:**
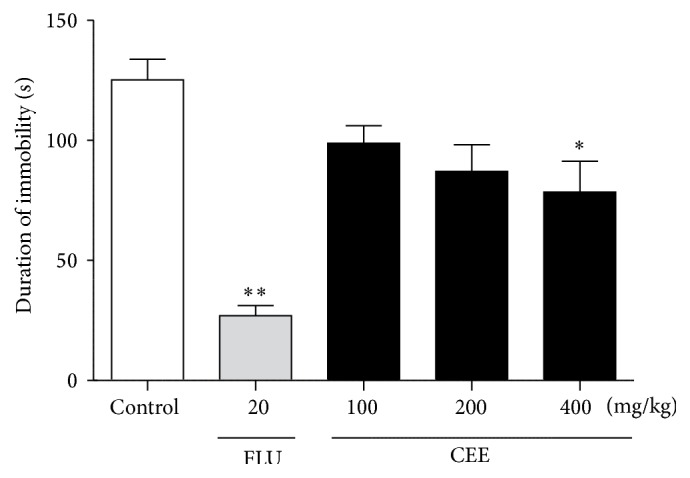
Effects of oral administration of CEE (100, 200, or 400 mg/kg) or fluoxetine (20 mg/kg) on the duration of immobility in the forced swimming test in mice. The total duration of immobility was recorded 1 h after the last administration. Values are presented as mean ± S.E.M. (*n* = 6 per group). ^*∗*^ *P* < 0.05, ^*∗∗*^ *P* < 0.01 as compared with vehicle control.

The antidepressant effect of CEE was investigated in the forced swimming test. The results in Figure 3 showed that, compared with the control group, CEE at a dose of 400 mg/kg significantly decreased the duration of immobility (*P* < 0.05), resulting in a 37.34% immobility reduction, while animals administrated with CEE at doses of 100 and 200 mg/kg demonstrated no statistically significant increase in the duration of immobility. Additionally, fluoxetine, a classical antidepressant agent, caused a significant reduction in the immobility time (*P* < 0.01) in the FST (78.47%).

### 3.5. Effects of CEE on the Duration of Immobility in the TST

The effects of CEE on the duration of immobility in the tail suspension test are shown in Figure 5. When compared with the control group, CEE at doses of 200 (*P* < 0.05) and 400 mg/kg (*P* < 0.01), as well as 20 mg/kg fluoxetine significantly (*P* < 0.01), shortened the duration of immobility in the TST by 48.32, 65.66, and 68.19%, respectively. The antidepressant effect of CEE at the highest dose (400 mg/kg) was similar to that of fluoxetine (20 mg/kg). However, the difference from the control group at the lowest dose (100 mg/kg) was not statistically significant.

### 3.6. Effects of CEE on Spontaneous Locomotor Activity

**Figure 4:**
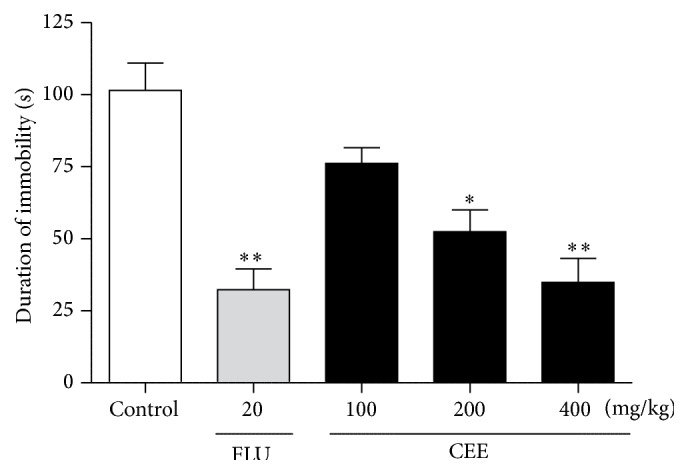
Effects of oral administration of CEE (100, 200, or 400 mg/kg) or fluoxetine (20 mg/kg) on the duration of immobility in the tail suspension test in mice. The total duration of immobility was recorded 1 h after the last administration. Values are presented as mean ± S.E.M. (*n* = 6 per group). ^*∗*^ *P* < 0.05, ^*∗∗*^ *P* < 0.01 as compared with vehicle control.

**Figure 5:**
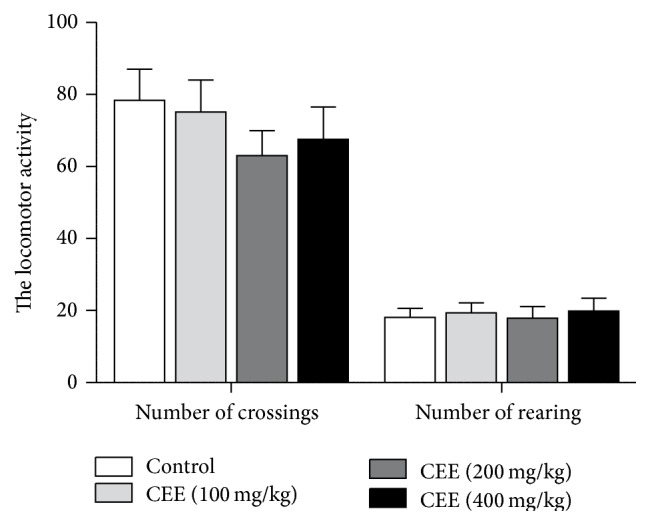
Effects of oral administration of CEE (100, 200, or 400 mg/kg) on the spontaneous locomotor activity in the open-field test in mice. Values are presented as mean ± S.E.M. (*n* = 6 per group).

In this test, treatments with CEE at doses of 100, 200, and 400 mg/kg exhibited no significant difference in the number of crossings and rearing as compared with the control group (Figure 4).

### 3.7. Effects of ASE on the Levels of Monoamine Neurotransmitter in Mice Brain

**Figure 6:**
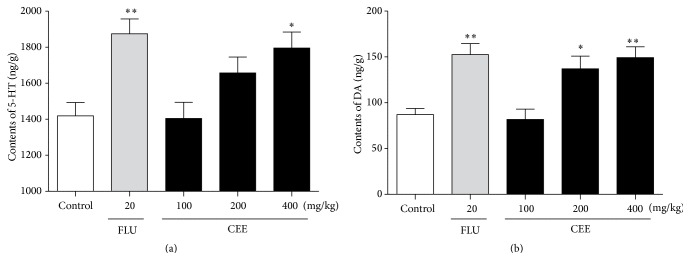
Effects of CEE (100, 200, or 400 mg/kg) or fluoxetine (20 mg/kg) on the contents of 5-HT and DA in the whole brain of mice following the tail suspension test. Values are presented as mean ± S.E.M. (*n* = 6 per group). ^*∗*^ *P* < 0.05, ^*∗∗*^ *P* < 0.01 as compared with vehicle control.

Results from the ELISA determination of 5-HT in mice brain were shown in Figure 6(a). The pretreatment of 400 mg/kg CE (*P* < 0.05) or 20 mg/kg fluoxetine (*P* < 0.01) significantly elevated the level of 5-HT as compared with the control group.  Figure 6(b) shows that the oral administration of CEE at 100 or 400 mg/kg significantly increased DA level in mice brain [*P* < 0.05, *P* < 0.01, resp.]. Moreover, in this test, the effects of CEE at dose of 100, 200, and 400 mg/kg presented a trend to increase after 7 consecutive days of treatment.

## 4. Discussion

The results of these experiments show for the first time that the aqueous extract of the leaves from* Camellia euphlebia* possesses anxiolytic effects as demonstrated in the light-dark box and elevated plus-maze test, as well as producing antidepressant activities during the forced swimming and tail suspension test.

The animal models mentioned above are considered as the most widely validated tests for assaying anxiolytic and antidepressant substances such as benzodiazepines or amine uptake inhibitors [28–30]. The light-dark box and elevated plus-maze test are the two most commonly used animal models of anxiety with which to screen anxiolytic drugs. A natural conflict between the tendency to explore and the initial tendency to avoid an unknown risk occurs when a mouse is exposed to an unfamiliar environment. The exploratory activity reflects the combined effects of these tendencies in novel situations. Thus, in the light-dark box test, drug-induced increases in time spent in the light box and is suggested as an index of anxiolytic activity [31]. Likewise, in the elevated plus-maze model, based on the principle that is exposure to an elevated and open arm maze leads to a conflict, while the number of open arm entries and time spent in the open arm provide a measure of anxiety-induced inhibition of the normal exploratory activity [32]. The forced swimming test and tail suspension test are two behavioral despair models which give an indication of the clinical efficacy of various types of antidepressant drugs in rodents. These animal models are based on the despair or helplessness behavior in response to some inescapable and confined space and are sensitive to various antidepressant drugs. The forced swimming and tail suspension-induced state of immobility in animals are claimed to represent a condition similar to human depression and are amenable to be reversed by antidepressant drugs [33].

The results obtained from the forced swimming test and the tail suspension test demonstrate that oral administration of CEE at a high dose of 400 mg/kg for 7 consecutive days produces a significant reduction in the duration of immobility, since the significant reduction of immobility time elicited by CEE cannot be attributable to any psychostimulant effect in the OFT. In addition, treatment with CEE at a low dose of 100 mg/kg significantly increased the time spent in the light box in the light-dark box test and the percentage of open arm entries and time spent in open arms in the elevated plus-maze test. This suggests that anxiety and depression show important overlaps, and even anxiety may be an early manifestation of depression as suggested by analyzing the obtained experimental results that mice treated with CEE at a low dose exhibited anxiolytic activities while they demonstrated characteristics of depression with a high dose, although anxiety and depression are considered as two different mental diseases. Moreover, 400 mg/kg CEE significantly elevated the level of 5-HT and DA. Such an observation suggests that the antidepressant effects of CEE may be caused by the preservation of monoamine neurotransmitters.

The active compounds present in the leaves of* Camellia euphlebia* are unknown and we cannot discard the possibility that more than one compound is responsible for its behavioral effects. Phytochemical studies have reported the presence of several compounds in the leaf extract of* Camellia euphlebia* including theanine (*γ*-glutamylethylamide), *γ*-aminobutyric acid (GABA), and caffeine [13]. The monoamine hypothesis of depression postulates a pathogenic role for disturbances in the monoaminergic system, involving not only norepinephrine, 5-hydroxytryptamine, and dopamine but also excitatory and inhibitory amino acid receptor families and second messengers [34]. Theanine (*γ*-glutamylethylamide or 5-N-ethylglutamine), a unique amino acid exclusively found in tea, has been reported to increase the level of the inhibitory neurotransmitters, primarily glycine, thus promoting dopamine release, which exert neuroprotective effects [35].

It was reported that the anxiolytic and antidepressant effects of caffeine are ascribed to its antagonistic properties at A1 and A2A adenosine receptors, which interact with other neurotransmitters and proteins [36]. In particular, antagonism of A2A receptors increases neurotransmission through dopamine D2 receptors, and antagonism of A1 receptors interacts with D1 receptors and regulates the release of neurotransmitters such as dopamine, glutamate, and acetylcholine [37, 38].

In addition, GABA is known as an inhibitory neurotransmitter present almost exclusively in the central nervous system, and GABAergic dysfunction causes mood disorders or neurological disorders such as seizures, anxiety, and depression [39]. Recent evidence has suggested the involvement of the GABAergic system in depression and in the mechanism of action of somatic antidepressant treatments [40]. In particular, GABA_B_ receptors have been found to be increased in the animal frontal cortex following chronic antidepressant therapies [41, 42]. Most strikingly, in patients with major depression, a reduced level of GABA was found in plasma and cerebrospinal fluid and in occipital cortical brain [43, 44]. Thus, a deficit in the GABAergic system seems to contribute to both anxiety and depression. Taken together, induced attenuation of GABAergic system by the GABA present in leaves of* Camellia euphlebia* seems to be part of the therapeutic effects in both anxiety and depression in mice.

It is speculated that the intracellular mechanism of depression involves the cAMP-CREB signal transduction pathway and Ca^2+^/calmodulin-dependent pathways. Antidepressant treatment has been reported to upregulate the cAMP-CREB cascade, including increased adenylyl cyclase, upregulation of cAMP-dependent protein kinase, and increased function and expression of CREB, which activates the mRNA expression of BDNF gene in the nucleus, thus contributing to synaptic remodeling and increased neurogenesis [45]. Additionally, CREB can be regulated by Ca^2+^-dependent protein kinases, which may be excessively activated by intracellular Ca^2+^ overloading [46]. On the basis of advances in molecular and cellular neurobiology to depression, further studies are needed to clarify the mechanism of action and to determine if administration of CEE is beneficial for patients with anxiety and depression.

## 5. Conclusions

Our data showed that CEE exerted anxiolytic and antidepressant effects in several classical animal model tests. Additionally, the results indicated that CEE is nontoxic when given acutely and may have a sedative effect at high doses. Overall, this study provides valuable preliminary data on the anxiolytic and antidepressant activities of* Camellia euphlebia* that should be useful for the planning of future preclinical and clinical studies of this plant medicine.

## Figures and Tables

**Table 1:** Effects of oral administration of CEE (500, 1000, 2000, or 4000 mg/kg) or normal saline (0.4 mL/25 g) on toxic symptoms and mortality in mice.

Drug	Dose (mg/kg)	Mortality (%)	Symptoms of toxicity^*∗*^
Saline	—	0	None
CEE	500	0	None
CEE	1000	0	None
CEE	2000	0	Sedation
CEE	4000	0	Sedation, hyperventilation

^*∗*^The toxic symptoms include sedation, hypoactivity, loss of appetite, piloerection, convulsion, dizziness, syncope, and hyperventilation.

## References

[1] World Health Organization (2001). *The World Health Report: 2001: Mental Health: New Understanding, New Hope*.

[2] Reynolds E. H. (2003). Brain and mind: a challenge for WHO. *The Lancet*.

[3] Whiteford H. A., Degenhardt L., Rehm J. (2013). Global burden of disease attributable to mental and substance use disorders: findings from the Global Burden of Disease Study 2010. *The Lancet*.

[4] Pacher P., Kecskemeti V. (2004). Cardiovascular side effects of new antidepressants and antipsychotics: new drugs, old concerns?. *Current Pharmaceutical Design*.

[5] Rothschild A. J. (2000). Sexual side effects of antidepressants. *Journal of Clinical Psychiatry*.

[6] Masand P. S., Gupta S. (2002). Long-term side effects of newer-generation antidepressants: SSRIS, venlafaxine, nefazodone, bupropion, and mirtazapine. *Annals of Clinical Psychiatry*.

[7] Jin L., Wu F., Li X. (2013). Anti-depressant effects of aqueous extract from acanthopanax senticosus in mice. *Phytotherapy Research*.

[8] Pawar V. S., Anup A., Baokar S., Shivakumar H. (2011). Antidepressant-like effects of *Acorus calamus* in forced swimming and tail suspension test in mice. *Asian Pacific Journal of Tropical Biomedicine*.

[9] Kim J.-H., Kim S. Y., Lee S.-Y., Jang C.-G. (2007). Antidepressant-like effects of *Albizzia julibrissin* in mice: involvement of the 5-HT_1A_ receptor system. *Pharmacology Biochemistry and Behavior*.

[10] Kalkunte S. S., Singh A. P., Chaves F. C. (2007). Antidepressant and antistress activity of GC-MS characterized lipophilic extracts of *Ginkgo biloba* leaves. *Phytotherapy Research*.

[11] Mao Q., Huang Z., Ip S., Che C. (2008). Antidepressant-like effect of ethanol extract from *Paeonia lactiflora* in mice. *Phytotherapy Research*.

[12] Butterweck V., Jürgenliemk G., Nahrstedt A., Winterhoff H. (2000). Flavonoids from *Hypericum perforatum* show antidepressant activity in the forced swimming test. *Planta Medica*.

[13] Lin J.-N., Lin H.-Y., Yang N.-S. (2013). Chemical constituents and anticancer activity of yellow camellias against MDA-MB-231 human breast cancer cells. *Journal of Agricultural and Food Chemistry*.

[14] Wan C.-P., Yu Y.-Y., Zhou S.-R., Cao S.-W. (2011). Antioxidant and free radical scavenging activity of *Camellia nitidissima* Chi. *Asian Journal of Chemistry*.

[15] Huang Y.-L., Chen Y.-Y., Wen Y.-X., Li D.-P., Liang R.-G., Wei X. (2009). Effects of the extracts from Camellia nitidssimas leaves on blood lipids. *Lishizhen Medicine and Materia Medica Research*.

[16] Xia X., Huang J.-J., Wang Z.-P., Wang Q., Pan W.-G. (2013). Hypolipidemic effect and acute toxicity of leaves from *Camellia nitidissima* Chi. *Lishizhen Medicine and Materia Medica Research*.

[17] Cho Y. R., Chang J. Y., Chang H. C. (2007). Production of *γ*-aminobutyric acid (GABA) by *Lactobacillus buchneri* isolated from Kimchi and its neuroprotective effect on neuronal cells. *Journal of Microbiology and Biotechnology*.

[18] Egashira N., Hayakawa K., Mishima K., Kimura H., Iwasaki K., Fujiwara M. (2004). Neuroprotective effect of *γ*-glutamylethylamide (theanine) on cerebral infarction in mice. *Neuroscience Letters*.

[19] Ritchie K., Carrière I., De Mendonça A. (2007). The neuroprotective effects of caffeine. A prospective population study (the Three City Study). *Neurology*.

[20] Miller L. C., Tainter M. L. (1944). Estimation of the ED50 and its error by means of logarithmic-probit graph paper. *Experimental Biology and Medicine*.

[21] Costall B., Domeney A. M., Gerrard P. A., Kelley M. E., Naylor R. J. (1988). Zacopride: anxiolytic profile in rodent and primate models of anxiety. *Journal of Pharmacy and Pharmacology*.

[22] Crawley J. N. (1985). Exploratory behavior models of anxiety in mice. *Neuroscience & Biobehavioral Reviews*.

[23] Carrasco M. C., Vicens P., Vidal J., Redolat R. (2006). Effects of co-administration of bupropion and nicotinic agonists on the elevated plus-maze test in mice. *Progress in Neuro-Psychopharmacology and Biological Psychiatry*.

[24] Hogg S. (1996). A review of the validity and variability of the elevated plus-maze as an animal model of anxiety. *Pharmacology Biochemistry and Behavior*.

[25] Porsolt R. D., Bertin A., Jalfre M. (1977). Behavioral despair in mice: a primary screening test for antidepressants. *Archives Internationales de Pharmacodynamie Et de Thérapie*.

[26] Steru L., Chermat R., Thierry B., Simon P. (1985). The tail suspension test: a new method for screening antidepressants in mice. *Psychopharmacology*.

[27] Campos A. R., Barros A. I. S., Albuquerque F. A. A., Leal L. K. A. M., Rao V. S. N. (2005). Acute effects of guarana (*Paullinia cupana* Mart.) on mouse behaviour in forced swimming and open field tests. *Phytotherapy Research*.

[28] Mora S., Díaz-Véliz G., Millán R. (2005). Anxiolytic and antidepressant-like effects of the hydroalcoholic extract from *Aloysia polystachya* in rats. *Pharmacology Biochemistry and Behavior*.

[29] Aragão G. F., Carneiro L. M. V., Junior A. P. F. (2006). A possible mechanism for anxiolytic and antidepressant effects of alpha- and beta-amyrin from *Protium heptaphyllum* (Aubl.) March. *Pharmacology Biochemistry and Behavior*.

[30] Reis J. S., Oliveira G. B., Monteiro M. C. (2014). Antidepressant-and anxiolytic-like activities of an oil extract of propolis in rats. *Phytomedicine*.

[31] Bourin M., Hascoët M. (2003). The mouse light/dark box test. *European Journal of Pharmacology*.

[32] Walf A. A., Frye C. A. (2007). The use of the elevated plus maze as an assay of anxiety-related behavior in rodents. *Nature Protocols*.

[33] Castagné V., Moser P., Roux S., Porsolt R. D. (2011). UNIT 8.10A Rodent models of depression: forced swim and tail suspension behavioral despair tests in rats and mice. *Current Protocols in Neuroscience*.

[34] Marazziti D., Rutigliano G., Baroni S., Landi P., Dell'Osso L. (2014). Metabolic syndrome and major depression. *CNS Spectrums*.

[35] Yamada T., Terashima T., Kawano S. (2009). Theanine, *γ*-glutamylethylamide, a unique amino acid in tea leaves, modulates neurotransmitter concentrations in the brain striatum interstitium in conscious rats. *Amino Acids*.

[36] Lara D. R. (2010). Caffeine, mental health, and psychiatric disorders. *Journal of Alzheimer's Disease*.

[37] Fredholm B. B., Bättig K., Holmén J., Nehlig A., Zvartau E. E. (1999). Actions of caffeine in the brain with special reference to factors that contribute to its widespread use. *Pharmacological Reviews*.

[38] Ferré S. (2008). An update on the mechanisms of the psychostimulant effects of caffeine. *Journal of Neurochemistry*.

[39] Möhler H. (2012). The GABA system in anxiety and depression and its therapeutic potential. *Neuropharmacology*.

[40] Luscher B., Shen Q., Sahir N. (2011). The GABAergic deficit hypothesis of major depressive disorder. *Molecular Psychiatry*.

[41] Pilc A., Lloyd K. G. (1984). Chronic antidepressants and GABA ‘B’ receptors: a GABA hypothesis of antidepressant drug action. *Life Sciences*.

[42] Pilc A., Nowak G. (2005). GABAergic hypotheses of anxiety and depression: focus on GABAB receptors. *Drugs of Today*.

[43] Sanacora G., Mason G. F., Rothman D. L. (1999). Reduced cortical *γ*-aminobutyric acid levels in depressed patients determined by proton magnetic resonance spectroscopy. *Archives of General Psychiatry*.

[44] Sanacora G., Mason G. F., Krystal J. H. (2000). Impairment of GABAergic transmission in depression: new insights from neuroimaging studies. *Critical Reviews in Neurobiology*.

[45] Duman R. S. (2002). Pathophysiology of depression: the concept of synaptic plasticity. *European Psychiatry*.

[46] Wu F., Li H., Zhao L. (2013). Protective effects of aqueous extract from *Acanthopanax senticosus* against corticosterone-induced neurotoxicity in PC12 cells. *Journal of Ethnopharmacology*.

